# Deterioration After Mild Traumatic Brain Injury: A Single-Center Experience With Cost Analysis

**DOI:** 10.3389/fneur.2021.588429

**Published:** 2021-09-24

**Authors:** Rafał Chojak, Marta Koźba-Gosztyła, Mateusz Pawłowski, Bogdan Czapiga

**Affiliations:** ^1^Faculty of Medicine, Wrocław Medical University, Wrocław, Poland; ^2^Department of Neurosurgery, 4th Military Hospital in Wrocław, Wrocław, Poland; ^3^Department of Nervous System Diseases, Faculty of Health Sciences, Wrocław Medical University, Wrocław, Poland

**Keywords:** deterioration, neurosurgical intervention, neurotraumatology, healthcare cost, mild TBI, neurosurgical observation, MTBI

## Abstract

**Background:** Most traumatic brain injuries (TBIs) are mild (GCS score of 13–15). Patients with mild TBI (mTBI) are generally in good condition. In some cases, a neurological deterioration (manifested by a drop of ≥1 point in GCS score) can occur and neurosurgical intervention (NI) may be needed. Because of that, these patients are frequently admitted to a hospital for observation. The aim of our study was to determine the number of patients with mTBI that deteriorate or need NI. We also considered an economic aspect of hospital admissions of these patients.

**Methods:** The study group consisted of 186 adult patients admitted to the neurosurgical department due to mTBI. Patients were divided into three groups according to an initial GCS score. The occurrence of deterioration, need for NI, length of stay (LOS), cost of stay and occurrence of death were analyzed.

**Results:** The deterioration was observed in 7 (3.76%) out of all cases. In 3 (1.61%) of them, the NI was needed. The average LOS was 7.96 days and it was closely linked with an initial GCS score (*p* < 0.001). The total cost of stay of all patients included in this study was about 1,188,668 PLN (306,357 USD).

**Conclusion:** The deterioration occurred in a small number of patients with mTBI, the need for NI was even less common. Hospitalization of these patients is expensive. Further studies with prognostic model helping decide on admission/discharge are necessary.

## Introduction

Traumatic brain injury (TBI) is a serious problem in majority of countries, which results in death and disability for thousands of people each year. The leading causes of TBI are falls and motor vehicle crashes. It is estimated that TBI is experienced by over 50 million people worldwide yearly. In Europe, over 1 million patients are hospitalized annually for TBI (1–3).

Conventionally, based on the Glasgow Coma Scale (GCS), TBI is classified as mild (GCS of 15–13), moderate (GCS of 12–9), and severe (GCS of 8–3) (4). The largest group of TBI patients, which is approximately 95%, are those with mild TBI (mTBI) (5). According to the American Congress of Rehabilitation Medicine, a patient with mTBI meets at least one of the following criteria: initial GCS score of 13–15, loss of consciousness immediately after accident lasting less than 30 min, post-traumatic amnesia lasting less than 24 h, and focal neurological deficits that may or may not be transient (6). Although mTBI patients are in good general condition with a high conscious level at presentation, approximately 12% of them will deteriorate and 3.5% will need neurosurgical intervention (7).

Despite many studies, there is still a lack of valid decision rules to manage mTBI patients. Some centers recommend hospital admission and observation for 24 h or more. Others suggest that low-risk patients can be discharged home. But criteria for low/high-risk mTBI patients are also unclear (8, 9). Prognostic models such as IMPACT or CRASH are useful for the group with moderate or severe TBI but not mild (10). In-hospital observation seems to be the best solution but hardly feasible and expensive, considering that the number of patients with mTBI varies from 302 to 600 per 100,000 people per year (11, 12).

## Aim of the Study

This study aimed to evaluate the frequency of 1) deterioration and 2) neurosurgical intervention (NSI) of patients with mTBI during their hospital stay. The second goal was to assess the length of stay (LOS) and the total cost of stay of these patients.

## Materials and Methods

### Patient Selection

We retrospectively analyzed the data of adult patients with head trauma admitted to the Neurosurgical Department in the 4th Military Hospital in Wroclaw between 2008 and 2019. We included only patients with GCS ≥ 13 based on their level of consciousness at the time of admission who were referred to the neurosurgical department for conservative treatment. All patients (1) with an initial GCS score below 13; (2) with subdural hematoma; (3) who were younger than 18 years old at admission; or (4) who were requiring emergency surgery were excluded from the study.

Then, we retrospectively categorized the patients into three groups based on their initial GCS score (13–15). The notes from medical records were evaluated for the information on the deterioration, length of stay, presence of alcohol, and mortality of patients during their hospital stay. Data on baseline patient characteristics were also retrieved.

### Deterioration and Decision for Neurosurgical Intervention

Deterioration was defined as GCS drop ≥ 1 in patients admitted to the neurosurgical department during their hospital stay. The decision for surgical intervention was made by neurological examination and CT scan.

### Cost Analysis

The total cost of stay was assessed based on the total LOS multiplied by the cost of in-patient day in our department (802.07 PLN in January 2020). This was about 206.72 USD (based on the exchange rate in 31 January 2020 from the National Polish Bank−3.88 PLN for USD).

### Statistical Analyses

Arithmetic means and standard deviations (SDs) were calculated for quantitative variables of the evaluated parameters in the studied groups. Variables are shown as percentages and numbers for categorical variables, and as means or medians with SD or ranges for continuous variables. The sample size was determined by the number of cases during the study period. In further statistical analysis, we tested the data for normality using a Shapiro test. The data were not normally distributed; thus, we used a nonparametric Kruskal–Wallis ANOVA test to investigate the effects of the initial GCS score on the LOS of patients. To determine the exact source of the differences, we used the Bonferroni correction to perform a pairwise comparison. Statistical analysis of the results was conducted using SPSS 24 (IBM SPSS Statistic, IBM Corporation). The significant difference was set at *P* < 0.05.

## Results

### Patient Characteristics

We screened 462 patients with head trauma, mostly due to fall or road traffic accident. Of them, we included 186 patients conservatively treated due to mTBI at the neurosurgical department. There were 44 women and 142 men. The mean age of included patients ranged from 19 to 90, with a mean of 58.5 ± 20.3 years. Forty patients were under the influence of alcohol at admission. Clinical features of 186 conservatively treated due to mTBI are summarized in Table 1.

**Table 1:** Characteristics of the study group.

**Admitted to hospital**	**186**
Gender (N, %)	
Female	44 (23.6%)
Male	142 (76.4%)
Mean age (years)	58.5 ± 20.3 (19–90 range)
Initial GCS (N, %)	
15	119 (64%)
14	52 (27.9%)
13	15 (8.1 %)
Presence of alcohol (N, % in group)	
15	18 (15.1%)
14	17 (32.7%)
13	5 (33.3%)

### Complications, Neurosurgical Intervention, and Mortality

Deterioration was observed in seven (3.76%) patients. In particular groups, there were three (2.52%) cases of deterioration in the GCS 15 group, two (3.85%) cases in the GCS 14 group, and two (13.33%) cases in the GCS 13 group. One (6.66%) patient in the GCS 13 group who deteriorated required NSI. In the GCS 14 group, two patients (3.85%) required NSI. In these cases, a decompressive craniectomy was performed. These three cases with the need for NSI represented 1.61% out of all patients included in this study. Both patients from the GCS 14 group successfully recovered after surgery. The patient from the GCS 13 group who deteriorated died 8 days after surgery (Table 2).

**Table 2:** Results of analysis in each GCS group.

**Initial GCS score**	**13**	**14**	**15**
	***N* = 15**	***N* = 52**	***N* = 119**
GCS drop	2 (13.33%)	2 (3.85%)	3 (2.52%)
NSI	1 (6.66%)	2 (3.85%)	0
Deaths	1	0	0

*GCS, Glasgow coma scale; NSI, Neurosurgery intervention; SD, standard deviation*.

### Length of Stay Cost Analysis

The total LOS at the neurosurgical department of all the patients included in the study was 1,482 days. The average LOS was 7.96 ± 7.2 days. The LOS was closely linked with the initial GCS score (*p* < 0.001). Patients with GCS of 15 had significantly shorter LOS compared with those with GCS 14 (*p* = 0.002) and 13 (*p* = 0.002). No significant differences were observed between the GCS 14 and 13 groups (*p* = 0.561).

The total cost of the stay for all patients was about 1,188,668 PLN (306,358 USD). The total cost of stay of 179 nondeteriorating patients was about 1,101,242 PLN (283,825 USD). The LOS and the costs of hospitalization in each group are presented in Table 3.

**Table 3:** Age, length of stay and cost of stay in all patients with mTBI regard to GCS score.

		**GCS score**			
	**13**	**14**	**15**	**Total**	
		***N* = 15**	***N* = 52**	***N* = 119**	***N* = 186**
Mean (SD)	Age	62.5 ± 19.1	59.5 ± 16.7	57.6 ± 21.9	58.6 ± 20.3
	LOS	12.3 ± 8.2	10.2 ± 8.5	6.4 ± 5.9	8.0 ± 7.2
Total	LOS	184	532	766	1,482
	Cost (PLN)^*^	147,581	426,701	614,386	1,188,668
	Cost (USD)^**^	38,036	109,975	158,347	306,358

*mTBI, mild traumatic brain injury; GCS, Glasgow coma scale; SD, standard deviation; LOS, length of stay (days).*

* *Cost per in-patient day (January 2020) was 802.07 PLN (without including procedures, medication etc.).*

** *USD exchange rate in 31.01.2020 from National Polish Bank-−3.88 PLN for USD*.

## Discussion

The present study highlights a low number of patients with mTBI who deteriorated during their hospital stay. Only 3 out of 186 patients included in our study required neurosurgical intervention. Our study also shows that an initial GCS is associated with LOS in these patients.

Of all patients with TBI, approximately 95% have an initial GCS of 13–15, indicating normal or mildly impaired responsiveness and orientation (5). The management of this large group of patients, with the so-called mild TBI (mTBI), remains controversial. Some centers advocate that all mTBI patients with changes in CT scans should be admitted to neurosurgical care with repeated CT scans (13). That policy generates extremely high costs in the world healthcare system. Other studies report that some low-risk patients may be safely discharged after a short period of observation in the ED (14, 15). The prognostic models that were made previously, such as IMPACT, TARN, or CRASH, are useful in predicting outcomes in patients with severe TBI rather than mild (10, 16).

In the meta-analysis of Marincovitz et al., the risk of clinical deterioration was 11.7%, neurosurgical interventions 3.5%, and death 1.4% (7). These values were higher as compared to the results of the present study. The authors of the Canadian CT Head Rule assessed that only 1% of mTBI patients required neurological intervention. They presented a list of intracranial injuries that would never require neurosurgical intervention (17). In the report from the United Kingdom, the authors underlined the low requirement for craniotomy and ICP monitoring in mild TBI, which was 3.8%. They concluded that transfer to a neurosurgical center for those patients may be unnecessary (18). In the recent publication of Marincowitz et al., the estimated prevalence of clinical deterioration was 27.7%, higher than previously reported. The authors explained this by differences in study design (19).

Currently, the studies are focused on a prognostic model that could help to define the mTBI patients who are safe to discharge. The main factors that affect the risk of deterioration in TBI patients are age, anticoagulation, and initial GCS. The studies also revealed that the presence of midline shift/mass effect is predictive of adverse outcomes. In contrast, patients with isolated subarachnoid hemorrhages (iSAHs) have the lowest risk for adverse outcomes (7). In the study of Marincowitz et al. the authors concluded that fully conscious patients, with no focal neurology deficits (GCS 15), not taking anticoagulant or antiplatelet medication, who have a single simple skull fracture or hemorrhage <5 mm (not cerebellar or brainstem) on the CT brain scan, and up to two extracranial bony or organ injuries, do not require hospital admission (risk score 0). That would make it possible to safely discharge 1 in 20 patients from ED who were routinely admitted for observation. This would significantly reduce global healthcare costs (19). In our study, the cost of hospitalization for 179 nondeteriorating patients was about 1,101,242 PLN (283,825 USD). Given the increasing incidence of falls and traffic accidents (20), the number of patients with TBI is likely to increase over the next few years.

This study is limited by being a retrospective analysis and single-institution study design, which may restrict the generalizability of our findings to the overall population of patients with mTBI due to inherent management and selection biases. Moreover, many mild TBIs might be underdiagnosed, which also limits the generalizability of the results. The sample size was relatively small; therefore, we did not study any predictors for clinical deterioration. Moreover, comparisons between groups were limited by unequal size, especially for a low number of patients in the GCS 13 group.

Our study showed a relatively small number of patients with mTBI who deteriorated after hospital admission and even fewer who needed neurosurgical intervention. The length of stay of these patients is associated with an initial GCS. The decision on admission or discharge of these patients needs an individual approach. From our experience, these patients are generally easy to manage; but their hospitalization is expensive and might be unnecessary in many cases. Therefore, strict criteria of admission and prognostic model dedicated to these patients are necessary.

## Data Availability Statement

The raw data supporting the conclusions of this article will be made available by the authors, without undue reservation.

## Ethics Statement

Ethical review and approval was not required for the study on human participants in accordance with the local legislation and institutional requirements. Written informed consent for participation was not required for this study in accordance with the national legislation and the institutional requirements.

## Author Contributions

MK-G, BC, RC, and MP designed the study. RC performed the analyses. RC and MP collected the data. RC and MK-G drafted the manuscript. BC and MK-G provided study supervision and critical revision. All authors critically reviewed the manuscript, read and approved the last version of this manuscript.

## Conflict of Interest

The authors declare that the research was conducted in the absence of any commercial or financial relationships that could be construed as a potential conflict of interest.

## Publisher's Note

All claims expressed in this article are solely those of the authors and do not necessarily represent those of their affiliated organizations, or those of the publisher, the editors and the reviewers. Any product that may be evaluated in this article, or claim that may be made by its manufacturer, is not guaranteed or endorsed by the publisher.
